# Understanding Cancer Through the Lens of Epigenetic Inheritance, Allele-Specific Gene Expression, and High-Throughput Technology

**DOI:** 10.3389/fonc.2019.00794

**Published:** 2019-08-21

**Authors:** Maxwell P. Lee

**Affiliations:** High Dimension Data Analysis Group, Center for Cancer Research, National Cancer Institute, National Institutes of Health, Bethesda, MD, United States

**Keywords:** epigenetics, cancer, allele, inheritance, therapy

## Abstract

Epigenetic information is characterized by its stable transmission during mitotic cell divisions and plasticity during development and differentiation. This duality is in contrast to genetic information, which is stable and identical in all cells in an organism with exception of immunoglobulin gene rearrangements in lymphocytes and somatic mutations in cancer cells. Allele-specific analysis of gene expression and epigenetic modifications provides a unique approach to studying epigenetic regulation in normal and cancer cells. Extension of Knudson's two-hits theory to include epigenetic alteration as a means to inactivate tumor suppressor genes provides better understanding of how genetic mutations and epigenetic alterations jointly contribute to cancer development. High-throughput technology has greatly accelerated cancer discovery. Large initiatives such as TCGA have shown that epigenetic components are frequent targets of mutations in cancer and these discoveries provide new insights into understanding cancer etiology and generate new opportunities for cancer therapeutics.

## Introduction

Epigenetics, first coined by Conrad Waddington in 1940s, was a conceptual model that describes the development process of forming a multicellular organism from a fertilized zygote (1). The concept had its root in the earlier studies in embryology and developmental biology. This epigenetic concept provided mechanisms that can bring about cellular changes in development and physiology but not involving changes of genetic materials. Although Mendel's work on genetic inheritance was well-recognized but the exact biochemical nature of genetic material was not known until a decade later when the double helix model of DNA was proposed in 1953 (2, 3). Following the discovery of the double helix structure of DNA, there was an explosion of studies to understand how DNA sequences were replicated and used as templates to synthesize mRNAs, and how mRNA sequences were translated to produce proteins, resulting in different cellular phenotypes and ultimately organism phenotypes (4). This was culminated as the central dogma of molecular biology in 1958 (5). A major focus of the biological research since that time was to elucidate the molecular mechanisms that underlie the differential gene expression programing in cellular differentiation in development, physiological response in daily activities, and pathological changes in diseases. The details emerging from these studies led to a general understanding of association among DNA methylation, gene expression, and physiological changes at the levels of organisms and cells. The contemporary definition of epigenetics proposed by Holliday stated that epigenetics is the study of gene expression changes during cellular differentiation and mitotic inheritance of cellular gene expression pattern, which doesn't involve changes in DNA sequence (6). The expanded view of epigenetics includes many phenomena that can't be explained by Mendelian inheritance. Some prominent examples are X-chromosome inactivation and genomic imprinting in mammal and position effect variegation in *Drosophila* (7–11). Indeed, it was the study of these non-mendelian phenomena that largely initiated the identification and characterization of the biochemical components of epigenetic machinery. The current view of epigenetic system consists of DNA methylation, histone acetylation and methylation and other posttranslational modifications, chromatin remodeling complexes, and non-coding RNAs (12–15). Together, these epigenetic components control gene expression and form the basis of epigenetic memory that can be transmitted through mitotic cell division without DNA sequence changes.

There are numerous excellent reviews on epigenetics and cancer epigenetics. A few are cited here (12–14, 16, 17). In this short review, I will focus on a few selected topics that capture some aspects of epigenetics and epigenetic regulation in cancer from the perspective of epigenetic stability vs. plasticity and from the perspective of the allele-specific gene expression.

## Epigenetic Inheritance and Plasticity

**Figure 1:**
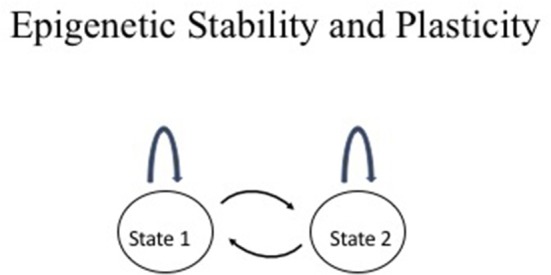
Epigenetic stability vs. plasticity. The two states may represent any two conceptual epigenetic states such as an active chromatin vs. an inactive chromatin state or normal cell vs. cancer cell. The arc above the state represents maintenance of the state through events such as mitotic division whereas the arrows between the two states represent interconversion between the two states such as changes in chromatin structure during cellular differentiation, physiological response, or disease.

Epigenetic information is characterized by its stable transmission during mitotic cell divisions and plasticity during development and differentiation. This duality differs from genetic information, which remains the same in every cell in an organism with the exception of a few cases such as immunoglobulin gene rearrangements in lymphocytes and somatic mutations in cancer. This duality is depicted in Figure 1. The two-states model provides a useful conceptual framework to think about epigenetic stability vs. plasticity.

Two classical examples of epigenetic phenomena are mammalian X chromosome inactivation and genomic imprinting (18, 19). Both are characterized by establishing active and inactive chromatin states in the two chromosomes in early embryogenesis, which are maintained during the life time of an organism. X chromosome inactivation involves gene expression silencing in one of the two X chromosomes, which ensures similar level of gene expression in both female and male cells. Which of the two X chromosomes to be inactivated is chosen randomly in the early embryogenesis. However, in the case of genomic imprinting, the inactivation occurs in specific genomic loci and the choice of which chromosome to be silenced is determined by the parental origin. Hundreds of imprinted genes have been identified. Some show gene silencing in all expressed tissues and during the entire life of the organism while others may display genomic imprinting only in selected tissues and affected by developmental stages and environmental exposure.

Studies of X chromosome inactivation and genomic imprinting played an instrumental role in establish DNA methylation and histone protein post-translational modifications and chromatin remodeling as the primary determinants of epigenetic state. There are many reasons why X chromosome and genomic imprinting are the excellent models to study epigenetics. The presence of a pair of active and inactive chromatin provides an ideal system to identify epigenetic marks that are specific for each epigenetic state but absent in the other epigenetic state. The DNA modification is relatively simple, involving methylation of the C5 in a cytosine (20). In mammalian genomes, the CpG dinucleotide occurs at much lower frequency than the other dinucleotides. This is because of the selective loss of CpG resulting from the conversion of 5-methylcytosine to thymine. However, there are genomic regions, where cluster of CpG dinucleotides are not methylated and consequently protected from the conversion, leading to the formation of CpG islands (CGIs) (21). About half of the mammalian genes contain CGIs, which are located near their transcription start sites.

Modifications of chromatin proteins are much more complex (12, 16). Both H3 and H4 histones undergo extensive post-translational modifications in their tails. These modifications include methylation, acetylation, phosphorylation, ubiquitination, etc. The combination of these modifications is referred to as the “histone code” (22), which carries the epigenetic information responsible for the maintenance of epigenetic state and dynamic change of epigenetic state.

From the perspective of epigenetic inheritance, DNA methylation state is maintained through DNA replication because semi-methylated DNA, the product of DNA replication, can be converted to fully methylated DNA by the action of DNMT1, which catalyze DNA methylation using semi-methylated DNA as substrate. DNA methyl transferase, DNMT3A, and DNMT3B, catalyze *de novo* methylation on DNA, thus providing a mechanism to acquire new DNA methylation marks to change chromatin state. However, the effort of searching for enzymes that can catalyze DNA demethylation was unsuccessful until about 10 years ago. It led to the thinking in the past that perhaps DNA demethylation could be mediated only by passively losing half of the methylation during each cycle of DNA replication in the absence of DNMT1 activity. This was changed recently, when it was discovered that Ten-eleven translocation (TET) enzymes can catalyze demethylation of 5-methycytosine through sequential conversion of 5-methycytosine to 5-hydroxymethyl cytosine, to 5-formylcytosine, then to 5-carboxylcytosine, which can be converted to unmodified cytosine by terminal deoxynucleotidyl transferase (TDT) (23, 24).

Histone modifications are far more complicated than DNA methylation. But the general strategy is similar. There exist a pair of enzyme systems, histone post-translational modification “writers” and “erasers.” For examples, histone acetyltransferase (HAT) serves as a writer whereas histone deacetylase (HDAC) serves as an eraser. Likewise, there are histone lysine methyltransferase (KMT) and histone lysine demethylase (KDM) to serve as writer and eraser, respectively. There are also protein arginine methyltransferases (PRMT), which act on arginine. Their opposing enzymes are peptidyl arginine deiminase (PADI). Each family also contains a large number of enzymes that can recognize specific substrate sequences. There is a third class of proteins called “readers” that can specifically bind to these post-translational modifications. For examples, bromodomain binds to acetylated lysine residue and chromodomain recognizes lysine methylation. Interestingly, histone acetyltransferase often contains bromodomain in addition to its activity to add acetylation to lysine. The multi-function structure of HAT enables it to catalyze acetylation in a processive manner to spread this post-translation mark (PTM) to the nearby nucleosome. This provides a potential mechanism for maintaining the PTM through mitotic division. Unlike DNA methylation, which produces semi-methylated DNA after DNA replication, the nucleosomes are randomly distributed into each of the two daughter cells after cell division. Half of the nucleosomes are derived from the parental cell and half are from newly deposited nucleosomes, which don't have PTMs. The ability of HAT to bind acetylated lysine and then catalyze addition of acetyl group to the nearby nucleosome allows the maintaining of this PTM through mitotic cell division.

The hallmark of epigenetics is the transmission of epigenetic marks through mitotic cell divisions. The duration of maintaining an epigenetic state varies. In the case of X chromosome inactivation and genomic imprinting, the active or inactive chromatin states are maintained throughout the lifetime. However, in most of cellular response to physiologic needs, the new epigenetic state is established and reversed back to normal state and the duration varies depending on particular physiology. DNA methylation mark is more stable while histone post-translational marks and other chromatin remodeling complexes display wide range of response time, serving different physiologic purposes.

## Allele-Specific Gene Expression

X chromosome inactivation and genomic imprinting are characterized by the mono-allelic gene expression and epigenetic modifications. Mono-allelic gene expression also occurs in a number of other biological systems. In B lymphocytes, once an immunoglobulin gene rearrangement takes place on one chromosome, the rearrangement of the same gene from the other chromosome would be prevented. This phenomenum is termed as allelic exclusion, which ensures that an individual lymphocyte expresses a unique amino acid sequence of an immunoglobulin protein (25). Similar mechanism also operates in T lymphocytes for activating TCR genes (26). Another example of mono-allelic expression is the expression of human olfactory receptor genes. There are about 1,000 olfactory receptor genes, each of which is expressed from only one chromosome in a sensory neuron (27).

In addition, quantitative differences in the degree of gene expression between two alleles, marked with SNPs, are a widespread phenomenon, hereinafter referred to as allele-specific expression (ASE). We initially studied allele-specific gene expression using the Affymetrix SNP arrays and found extensive allelic variation in expression in the human genome (28). ASE differs from mono-allelic expression described in the previous sections. ASE showed differential gene expression between the two alleles in the range of 2–4 fold, which is in contrast to mono-allelic expression observed in imprinting and X chromosome inactivation. ASE is commonly affected by genetic polymorphisms near the gene and these polymorphisms play a regulatory role affecting gene expression (29, 30). This is particularly relevant since most of the GWAS identified SNPs are located in intragenic or intergenic regions. These SNPs impact phenotypes through gene expression regulation at the epigenetic level or post-transcriptional level.

## Understanding Cancer From the Perspective of Epigenetic Regulation and Allele-Specific Gene Expression

It is well-established that cancer is caused by mutations that are acquired either from parents through germline inheritance or generated in somatic cells. Inactivating tumor suppressor genes and activating oncogenes both contribute to cancer development. In the case of inactivation of tumor suppressor genes, both alleles have to be inactivated in the cancer cell. This is best illustrated by Knudson's two-hits theory (31). The two-hits theory was postulated to explain why familial retinoblastoma develops earlier and bilateral while the sporadic retinoblastoma develops later and often unilateral. Based on the epidemiologic observation, Knudson hypothesized that retinoblastoma was caused by inactivation of both alleles of a tumor suppressor gene and in the case of familial syndrome one allele was inactivated in germline and the 2nd allele was inactivated in somatic tumor whereas in the case of sporadic cancer both alleles were inactivated in the somatic tumor. This paradigm can extend to include epigenetic alteration as a means to inactivate tumor suppressor genes. Many tumor suppressor genes, such as BRCA1/2 and CDKN2A/B, are frequently silenced by DNA methylation and inactive chromatin marks (32–34).

Epigenetic alteration through germline inheritance can occur in familial cancer syndrome. Lynch syndrome is caused by mutations in the genes involved in DNA mismatch repair. Germline mutations in MLH1 and MSH2 causes the majority of Lynch syndrome. However, in several Lynch syndrome families, no mutations in mismatch repairs genes were found despite extensive effort of searching for causative mutations. Instead, heritable DNA methylation in the promoter regions of MLH1 or MSH2 was identified that silenced gene expression. Chan et al. analyzed a three-generation family using allele-specific methylation (ASM) and reported that methylation of MSH2 gene in the germ line cells correlated with the loss of the MSH2 protein in the colorectal adenocarcinomas (35). Besides silencing gene expression by DNA methylation, a somatic frameshift mutation was found in MSH2. The authors concluded that ASM in germline transmission was the first hit while the somatic mutation was the 2nd hit. In a separate study of a HNPCC family, the EPCAM gene had a germline deletion in the 3′ end and resulted transcription read-through into downstream MSH2 and an increase in DNA methylation in the promoter region of MSH2 (36). The deletion was co-segregated with ASM of the MSH2 promoter and the disease. Epimutation of the RB1 gene was recently found in a six generations retinoblastoma family (37). The germline methylation was inherited from the maternal chromosome. Interestingly, the detailed pedigree analysis also found a germline mutation that was transmitted through the paternal chromosome and showed incomplete penetrance. The authors concluded that both genetic mutation and epimutation contributed to the retinoblastoma in this family. A rare epimutation in the RB1 gene was also identified from another recent study (38). The authors showed that germline DNA methylation was associated with silencing of the RB1 gene expression.

Beckwith–Wiedemann syndrome (BWS), is another familial syndrome, which increases risk of developing multiple pediatric cancers. It has served as a model system for studying genomic imprinting and how abnormal genomic imprinting causes cancer. Multiple genetic and epigenetic mechanisms were identified that cause BWS, including mutation in CDKN1C (39), loss of imprinting in IGF2 (40), translocation involving KCNQ1 (41), and abnormal imprinting of a lincRNA, KCNQ1OT1 (42). All four genes are imprinted. IGF2 is normally expressed from the paternal chromosome but expressed from both chromosomes in tumors. KCNQ1OT1 is normally methylated on maternal chromosome but the methylation is frequently lost in BWS patient germline DNA. Allele-specific gene expression and allele-specific methylation analysis have played an instrumental role in elucidating various epigenetic mechanisms (43).

Two papers brought about wide appreciation of quantitative difference in gene expression between two alleles of APC in familial adenomatous polyposis (FAP) (44, 45). Yan et al. showed that 50% reduction in gene expression in APC was associated with predisposition to FAP. They studied six patients from two FAP families and didn't find any mutation in the APC gene. However, using ASE, they found 2-fold difference in gene expression between the two alleles in all 6 patients. Furthermore, tumors displayed loss of heterozygosity (LOH) and deleted specifically the high-expression allele.

An interesting question is whether DNA methylation causes inactive chromatin state or vice versa. An elegant study from Vogelstein's lab provided an important insight into answering this question (46). They generated double knockout of DNMT1 and DNMT3B, which eliminated most of DNA methylation in HCT116, a colon cancer cell line. The double knockout cells had slow growth rate in early passage cells but the late passage cells grow to comparable rate to the parental cells. This corresponded to a gradual increase in p16 methylation and consequently silencing of p16. The kinetics of chromatin marks changes was faster. Histone H4-acetylation increased as early as the passage 5 and H3K9-methylation appeared at the passage 22. But DNA methylation appeared at the passage 50. The p16 is heterozygous in HCT116, allowing tracking of both alleles for allele-specific analysis of gene expression, DNA methylation, and chromatin marks. The study revealed that only the wild type allele showed dynamic changes of epigenetic marks. These observations established that the order of epigenetic changes in this system was that it began with the gain of H4-acetylation and expression of p16, followed by H3K9 methylation and silencing of p16 expression and faster growth, and eventually cells fully re-gained DNA methylation and lose H4-acetylation mark and grew at comparable rate as the parental cells. The work also demonstrated the important role of silencing p16 in driving cellular proliferation.

## Accelerating Cancer Discovery With High-Throughput Technology

The high-throughput analysis of gene mutations in human cancer was made possible after the human genome sequencing was completed in 2003. Some of the earliest studies that leveraged human genome sequence data to systematically identify mutated genes in human cancer were reported by the researchers from Johns Hopkins and Sanger Institute. These included the large scale analysis of coding sequences of human transcriptome in breast and colorectal cancer from Johns Hopkins in 2006 and 2007 (47, 48) and analysis of the coding exons of 518 protein kinase genes in multiple human cancers from Sanger Institute in 2007 (49). In 2005, NCI and NHGRI initiated The Cancer Genome Atlas (TCGA) initiative to comprehensively characterize genomic alterations in all major cancers. The pilot project was initially focused on glioblastoma multiforme (GBM) and ovary cancer, and it was extended to include more than 30 types of cancer in 2010. The first paper published from the TCGA initiative was the comprehensive analysis of mutation, DNA methylation, and gene expression of GBM in 2008 (50).

A very interesting study came from the comprehensive analysis of mutation in glioblastoma multiforme (GBM) by the Hopkins team in 2008, which led to the discovery of a recurrent mutation R132H in isocitrate dehydrogenase 1 (IDH1) and the mutation was shown to be associated with better survival (51). The R132H mutation was always present as a heterozygous mutation, suggesting it functions as an oncogene. Detailed biochemical study showed that the IDH1 mutation generated 2-hydroxyglutarate (2HG) instead of alpha-ketoglutarate, which is the normal product of the wildtype enzyme (52). Excessive accumulation of 2HG contributed to the formation of gliomas, suggesting that 2HG acted as an onco-metabolite.

In 2010, TCGA team found that a subset of GBM has high CpG island methylation, which was termed as a glioma CpG island methylator phenotype (G-CIMP) (53). G-CIMP tumors were more prevalent among lower-grade gliomas and associated with IDH1 somatic mutations. The association between DNA methylation and IDH1 was not unique to GBM and it also occurred in acute myeloid leukemia (AML) (54). What was really intriguing was the finding that mutations in IDH1/2 and TET2 were mutually exclusive in AML. TET2 was known to catalyze the conversion of 5 methyl cytosine to 5 hydroxy methyl cytosine. This immediately suggested that IDH mutation inhibits TET2 activity. Indeed, expression of IDH1 mutant inhibited the production of 5 hydroxy methyl cytosine. The mechanism for the inhibition was because IDH mutants produced 2-hydroxyglutarate instead of 2-oxoglutarate. 2-oxoglutarate was co-factor for TET2 to catalyze hydroxy methylation whereas 2-oxoglutarate served as a competitive inhibitor to TET2. Therefore, either Tet2 mutation or IDH2 could cause accumulation of 5 methyl cytosine, generating the CpG island methylation phenotype. This explained why either IDH1/2 or TET2 mutation could block hematopoietic differentiation and cause proleukemogenic effect.

One of the emerging concepts from the high-throughput mutational analysis of human cancer genomes was the finding that chromatin components are the frequent targets of mutations in human cancer (55–57). Some examples are provided here. Recurrent mutations of the histone methyltransferase MLL2 were detected in 89% of follicular lymphoma (FL) and 32% of diffuse large B-cell lymphoma (DLBCL) (58). The histone H3K27 demethylase UTX was mutated in multiple human cancers (59). Mutations in EZH2, a histone H3K27 methyltransferase, was found in GCB subtype of DLBCL and follicular lymphoma (60). Mutations in DNA methyltransferase DNMT3A were identified in 25% of acute myeloid leukemia (AML) (61). Not only the histone modifiers were frequently mutated, but histone proteins were also the direct targets of mutations in human cancer. Both histone H3 variant H3.3 (H3F3A) and the histone H3.1 (HIST1H3B) were mutated in 30% of pediatric glioblastomas (62, 63). Interestingly, these mutations occurred at the specific sites, K27M and G34R/G34V, and were present in heterozygous. Detailed biochemical studies showed that H3K27M mutant acted in a dominant-negative manner to inhibit PRC2 activities and consequently reduced H3K27me3 level (64).

## Epigenetic Dynamics in Cancer Treatment

DNA methyltransferase inhibitors, 5-azacytidine (Vidaza) and 5-aza-2′-deoxycytidine (decitabine), are FDA approved drugs for myelodysplastic syndrome (MDS) and AML patients. Treatment with 5-azacitidine and decitabine increased overall survival in MDS patients than conventional care in phase III clinical trials (65, 66). The response rates are between 30 and 60%. The response in myeloid malignancies are better than lymphoid leukemia or solid tumors (67). This might be related to the observation that myeloid leukemia has a relatively low mutational burden but has mutations in the genes involved in controlling DNA methylation, such as TET2 or DNMT3A (68). Many studies were conducted to understand what the clinical factors and molecular alterations are associated with treatment response, only TET2 mutation was found to be weakly associated with clinical response to therapy (69, 70). DNA methyltransferase inhibitors have bi-modal activities. At low dose, they cause hypomethylation whereas at high dose, they are cytotoxic. Following treatment, there was global decrease in DNA methylation and hypomethylation was associated with better response (71). Hypomethylation of specific tumor suppressor genes such as CDKN2B was also observed, which was associated with reactivation of protein expression to a normal level (72). This is consistent with the mechanism of drug action.

Vorinostat (SAHA), belinostat (PXD101), and romidepsin are FDA approved histone deacetylase (HDAC) inhibitors for cutaneous T cell lymphoma (CTCL) patients (73). The response rates are between 30 and 40% of patients with CTCL (74–76). Panobinostat in combination with the proteasome inhibitor bortezomib is FDA approved for the treatment of drug-resistant multiple myeloma (77). However, the success of these drugs is limited to cutaneous T cell lymphoma and multiple myeloma, and they are not effective for solid tumors. Similar to DNA methyltransferase inhibitors, HDAC inhibitors also show bi-modal activities. Their efficacy is dose-dependent, and the drugs are cytotoxic at high dose (78). The drug targets are more complex since there are eleven HDACs and also many non-histone targets. The targets could be nuclear or cytoplasmic. The complexity makes it hard to predict what factors could determine how well patients respond to treatment.

Besides targeting DNA methyltransferases and histone deacetylase, recently identified mutations in histone modifiers and chromatin remodeling proteins offer new opportunities for targeted therapy (55, 79). These include development of JQ1 and I-BET that bind to acetyl lysine recognition motifs of bromodomain and extra-terminal (BET) of BRD4 (80, 81), which is involved in DNA translocation in several cancers and activation of MYC oncogene; development of an inhibitor of H3K79 *N*-methyltransferase (DOT1L), which is involved in leukemogenesis in mixed lineage leukemia (MLL) (82); development of a small molecule GSK2879552 that inhibits lysine demethylase 1 (LSD1) (83).

A major concern in cancer therapy, either chemotherapy or targeted therapy, is the development of resistance to cancer drugs. Many mechanisms contribute to drug resistance, including drug efflux and mutations in the targeted genes or related pathways. However, recent studies suggested that epigenetic alterations could provide another mechanism to acquire drug resistance, especially for slowly acquired resistance (84). The drug resistance involved activation of IGF1 signaling pathway and chromatin alteration mediated by the histone demethylase KDM5A in a small population of drug-tolerant cells. Treatment with IGF1 receptor inhibitors or HDAC inhibitors can eliminate the drug-tolerant cells. The combination of chemo with HDAC inhibitors provide a potential new strategy to prevent development of drug resistance.

In conclusion, the studies of epigenetics and allele-specific gene expression and application of high-throughput technology provide powerful approaches to enhancing our understanding of cancer etiology and progression and also provide new opportunity for cancer therapeutics. There are some limitations when we try to understand cancer through the lens of epigenetic inheritance, allele-specific gene expression, and high-throughput technology. Germline epimutations are very rare events and some of which may be caused by yet unknown genetic variants. Although allele-specific gene expression can provide a unique perspective on the role of genetic variants on gene expression regulation, we are often more interested in the combined gene expression contributed from both alleles and how the gene expression is associated with other biological phenomena. High-throughput technology is powerful for the discovery phase of the research. However, new findings should be rigorously validated by additional experiments.

## Author Contributions

The author confirms being the sole contributor of this work and has approved it for publication.

### Conflict of Interest Statement

The author declares that the research was conducted in the absence of any commercial or financial relationships that could be construed as a potential conflict of interest. The reviewer JZ declared a shared affiliation, though no other collaboration, with the author to the handling editor.
